# Salidroside Attenuates High-Fat Diet-Induced Nonalcoholic Fatty Liver Disease via AMPK-Dependent TXNIP/NLRP3 Pathway

**DOI:** 10.1155/2018/8597897

**Published:** 2018-07-22

**Authors:** Tao Zheng, Xiaoyan Yang, Wenjin Li, Qibin Wang, Li Chen, Dan Wu, Fang Bian, Shasha Xing, Si Jin

**Affiliations:** ^1^Institute of Geriatric Medicine, Liyuan Hospital, Tongji Medical College, Huazhong University of Science and Technology, Wuhan, Hubei, China; ^2^Department of Pharmacy, Taihe Hospital, Hubei University of Medicine, Shiyan, Hubei, China; ^3^Hubei Key Laboratory of Drug Target Research and Pharmacodynamic Evaluation, Department of Pharmacology, School of Basic Medicine, Tongji Medical College, Huazhong University of Science and Technology, Wuhan, Hubei, China; ^4^Taihe Hospital, School of Medicine, Xi'an Jiaotong University, Shiyan, Hubei, China; ^5^Department of Endocrinology, Liyuan Hospital, Tongji Medical College, Huazhong University of Science and Technology, Wuhan, Hubei, China

## Abstract

Our previous studies suggested that salidroside could alleviate hepatic steatosis in type 2 diabetic C57BLKS/*Lepr^db^* (*db/db*) mice. The aim of the present study was to evaluate the therapeutic effect of salidroside on high-fat diet- (HFD-) induced nonalcoholic fatty liver disease (NAFLD) by investigating underlying mechanisms. Mice were fed with HFD or regular diet, randomly divided into two groups, and treated with salidroside or vehicle for 8 weeks. Then, biochemical analyses and histopathological examinations were conducted *in vivo* and *in vitro*. Salidroside administration attenuated HFD-induced obesity, blood glucose variability, and hepatic lipid deposition, markedly increasing insulin sensitivity in HFD mice. In addition, salidroside suppressed oxidative stress, thioredoxin-interacting protein (TXNIP) expression, and NLRP3 inflammasome activation in the liver. In cultured hepatocytes, salidroside dose dependently regulated lipid accumulation, reactive oxygen species (ROS) generation, and NLRP3 inflammasome activation as well as improved AMP-activated protein kinase (AMPK) activity and insulin sensitivity. The inhibition of AMPK activation by inhibitor or short interfering RNA (siRNA) resulted in the suppression of the beneficial effects of salidroside in hepatocytes. Our findings demonstrated that salidroside protects against NAFLD by improving hepatic lipid metabolism and NLRP3 inflammasome activation, and these actions are related to the regulation of the oxidative stress and AMPK-dependent TXNIP/NLRP3 pathways.

## 1. Introduction

Modern global health challenges are different from past generations, being often related to the increasing worldwide prevalence of obesity-related diseases, such as the nonalcoholic fatty liver disease (NAFLD) [1]. NAFLD seems to be a major public health concern not only in Western countries but also in the Asia-Pacific region [2, 3].

Patients presenting with NAFLD almost universally have insulin resistance, which increases the risk of type 2 diabetes [1, 4]. Previous studies demonstrated that more than 90% of obese patients with type 2 diabetes have NAFLD [5]. As one of the manifestations of metabolic syndrome, many patients with NAFLD remain asymptomatic, whereas 20% progress to develop nonalcoholic steatohepatitis (NASH) [6]. However, factors leading to the progression from NAFLD to NASH remain poorly understood, despite its high prevalence [6, 7]. A “two hit” mechanism has been proposed to drive NAFLD to NASH pathogenesis [6]. The proposed first hit was the accumulation of lipids within hepatocytes, and it is closely associated with mitochondrial abnormalities induced by lipotoxicity, which sensitize the liver to additional proinflammatory insults. The proposed second hit was a multifactorial process involving a combination of reactive oxygen species (ROS), lipid peroxidation, and proinflammatory cytokines [6, 8]. Obviously, the role of inflammation is not limited to the progression from NAFLD to NASH, but it is also deeply involved in the pathological process of chronic metabolic diseases, such as obesity, insulin resistance, and type 2 diabetes [9, 10].

Inflammasomes are cytoplasmic multiprotein complexes composed of one of several NOD-like receptors (NLRs) and PYHIN proteins, including NLRP1, NLRP3, NLRC4, and AIM2 [6]. Inflammasomes are sensors of endogenous or exogenous pathogen-associated molecular patterns (PAMPs) or damage-associated molecular patterns (DAMPs) that govern the production of IL-1*β* and IL-18 [10]. The most characterized member of the NLR family is the NLRP3 inflammasome [11]. Several reports demonstrated that the activation of NLRP3 inflammasome is associated with the pathological process of NAFLD [6, 12, 13]. ROS result as increased in the NAFLD pathogenesis and one of the major mammalian antioxidant system involves the thioredoxin- (TRX-) dependent peroxidase peroxiredoxin [14]. TRX-interacting protein (TXNIP), the endogenous negative regulator of cellular TRX that blocks its antioxidative function, may dissociate from TRX to bind with NLRP3 leading to the inflammasome activation [15–18].

Salidroside, a phenylpropanoid glycoside compound [2-(4-hydroxyphenyl)-ethyl-*β*-D-glucopyranoside], is the main active ingredient of *Rhodiola rosea*, a precious plant growing at high altitude zones that has been used for hundreds of years as traditional medicine for treating high altitude sickness [19]. Multiple evidences suggested that salidroside has the potential for the treatment of diseases related to the cardiovascular system [20, 21] or nervous system [22–24]. Our previous studies have clarified that salidroside has pharmacological properties including antioxidation and metabolic regulation and exerts therapeutic effects on type 2 diabetes [25, 26] and atherosclerosis [27, 28] via the activation of the mitochondria-associated AMP-activated protein kinase- (AMPK-) related signaling pathways. We found that lipid accumulation in the liver was substantially reduced after salidroside administration in C57BLKS/*Lepr^db^* (*db/db*) mice [25], and thus we speculated that salidroside may exert beneficial effects on NAFLD. However, molecular mechanisms underlying the protective actions of salidroside against NLFLD are not yet understood.

In the present study, we investigated the effects of salidroside on NAFLD by exploring the underlying mechanisms both *in vivo* and *in vitro*.

## 2. Materials and Methods

### 2.1. Animals

Male C57BL/6 mice (6 weeks old) were purchased from the Experimental Animal Center of Tongji Medical College, Huazhong University of Science and Technology (Wuhan, China). All the experimental procedures were performed in accordance with the International Guidelines for Care and Use of Laboratory Animals and approved by the Animal Ethical Committee of Tongji Medical College, Huazhong University of Science and Technology. The animals were housed at 22 ± 2°C, 45–75% relative humidity, and 12 h light-dark cycle. Before the experiment, the mice were kept for 1 week to acclimatize to the conditions. After acclimated, mice were fed either a regular diet (RD) or a high-fat diet (HFD) (21% fat, 0.15% cholesterol) for 14 weeks and then randomly divided HFD-mice into two groups. Mice were treated with vehicle (0.9% saline) or 100 mg·kg^−1^·d^−1^ salidroside (number 10338-51-9, purity > 98%, Tauto Biotech, Shanghai, China) for the next 8 weeks. Mice were anaesthetized by intraperitoneal injection of 1 mg·kg^−1^·body weight urethane (number 301912293, Sinopharm Chemical Reagent, Shanghai, China) and then sacrificed after blood samples and liver tissues were collected. For insulin signaling transduction analysis, mice were stimulated with 0.75 mU·g^−1^ insulin for 10 min after 12 h fasting before being sacrificed, and then liver tissues were collected and prepared for immunoblot analysis. Blood samples and liver tissues of *db/db* mice were used for biochemical analysis or immunoblot analysis in the current study, and the administration of *db/db* mice was described in our previous report [25].

### 2.2. Measurements of Postprandial Blood Glucose, Body Weight, and Serum Insulin Levels

Postprandial blood glucose and body weight of mice were monitored weekly during the period of salidroside treatment. For the measurement of postprandial blood glucose levels, food was removed for 2 h as previously described [29], then blood samples from the tail vein were collected and the glucose levels were measured by a blood glucose meter (LifeScan Inc., Milpitas, CA). Serum insulin levels and the homeostasis model assessment-insulin resistance (HOMA-IR) index were determined as previously described [30].

### 2.3. Intraperitoneal Glucose Tolerance Test (IPGTT) and Intraperitoneal Insulin Tolerance Test (IPITT)

IPGTT and IPITT were performed in the morning on nonfasted mice as previously described [29]. Briefly, after removing all food for 1 h, blood glucose levels were measured as noted above, then glucose (1 mg glucose per g body weight) or insulin (1 mU insulin per g body weight) was injected intraperitoneally. After injection, blood glucose levels were measured at 15, 30, 60, and 120 min.

### 2.4. Biochemical Analyses

The total cholesterol (TC), triglycerides (TG), HDL cholesterol (HDL-C), LDL cholesterol (LDL-C), nonesterified fatty acid (NEFA), aspartate transaminase (AST), alanine transaminase (ALT), malondialdehyde (MDA), and SOD levels in serum were determined by using commercial kits according to the manufacturer's instructions. TC and TG contents in liver tissues were analyzed as previously reported [31], and final results were normalized to those of weights in the initial homogenate. The TC (number 2401145), TG (number 2401144), LDL-C (number 2400321), and HDL-C (number 2401115) kits were purchased from Biosino Bio-Technology and Science Inc. (Beijing, China), while the NEFA (number A042-1), AST (number C010-2), ALT (number C009-2), MDA (number A003-1), and SOD (number A001-1) kits were purchased from Nanjing Jiancheng Bioengineering Institute (Nanjing, China). The serum IL-1*β* level was measured using an ELISA kit (number 70-EK2195) purchased from MultiSciences Biotech (Hangzhou, China) according to the manufacturer's instructions.

### 2.5. Histological Analysis of the Liver

The excised livers were rapidly cleaned and fixed in 10% neutral-buffered formalin solution. After embedding in paraffin, blocks were cut into 5 *μ*m thick sections and stained with hematoxylin-eosin (H & E). For the immunohistochemical analysis, paraffin sections were stained with CD68 antibody (number BA3638, Boster Biotechnology, Wuhan, China). For Oil Red O staining, liver tissues were fixed and dehydrated in 30% sucrose solution at room temperature and then were immersed in the optimal cutting temperature solution for procedures of Oil Red O (number O0625, Sigma-Aldrich, St. Louis, MO, USA) staining. The steatosis, inflammation, ballooning, and NAFLD activity were scored based on the NAFLD activity score system as previously described [32].

### 2.6. RNA Extraction and Quantitative Real-Time PCR

For the quantitative *real-time* PCR (qRT-PCR) analysis, total RNA was extracted from liver tissues by using the TRIzol reagent (number 15596-018, Invitrogen, CA, USA), then first strand cDNAs were synthesized using the RevertAid first strand cDNA synthesis kit (number K1622, Thermo Scientific, Sankt Leon-Rot, Germany). The qRT-PCR procedure was performed by using a SYBR Green qPCR kit (number QPK-201T, TOYOBO, Osaka, Japan) and ABI PRISM 7700 Sequence Detection System. Primer sequences used are listed as follows: *fatty acid synthase* (*Fas*)-forward, 5′-AGCGGCCATTTCCATTGCCC-3′, *Fas*-reverse, 5′-CCATGCCCAGAGGGTGGTTG-3′; *monocyte chemoattractant protein 1* (*Mcp1*)-forward, 5′-AGGTCCCTGTCATGCTTCTG-3′, *Mcp1*-reverse, 5′-TCTGGACCCATTCCTTCTTG-3′; *tumor necrosis factor α* (*Tnfα*)-forward, 5′-ATGAGCACAGAAAGCATGATC-3′, *Tnfα*-reverse, 5′-TACAGGCTTGTCACTCGAATT-3′; *Nlrp3*-forward, 5′-AGCCTTCCAGGATCCTCTTC-3′, *Nlrp3*-reverse, 5′-CTTGGGCAGCAGTTTCTTTC-3′; *caspase1*-forward, 5′-AGATGGCACATTTCCAGGAC-3′, *caspase1*-reverse, 5′-GATCCTCCAGCAGCAACTTC-3′; *Il1β*-forward, 5′-TCTTTGAAGTTGACGGACCC-3′, *Il1β*-reverse, 5′-TGAGTGATACTGCCTGCCTG-3′; *Il18*-forward, 5′-CAGGCCTGACATCTTCTGCAA-3′, *Il18*-reverse, 5′-TCTGACATGGCAGCCATTGT-3′; *β-actin*-forward, 5′-AAATCGTGCGTGACATCAAA-3′, *β-actin*-reverse, 5′-AAGGAAGGCTGGAAAAGAGC-3′. Expression levels were normalized to *β-actin*.

### 2.7. Cell Culture and Treatments

Primary hepatocytes were isolated from C57BL/6 mice as previously described [25]. Hepatocytes were plated into collagen-coated 6-well plates or 35 mm dishes (5 × 10^5^ per well or dish) in DMEM containing 5.5 mM glucose and 10% FBS. Cells were grown up to 70% confluence, and then the medium was changed with DMEM containing high glucose (30 mM) plus insulin (100 nM) after serum starvation. According to previous reports [25, 33], high glucose plus insulin treatment could mimic insulin resistance *in vivo* and cause superfluous lipid deposition in cultured hepatocytes. After treatment with or without salidroside, cell viability assay, lipid content and ROS level measurement, and immunoblotting were carried out. For assessment of insulin sensitivity *in vitro*, hepatocytes were treated as indicated above, the medium was removed, and cells were incubated in fresh serum-free DMEM containing 10 nM insulin (number I5500, Sigma-Aldrich, St. Louis, MO, USA) for 20 min before protein sample extraction. For the exploration of pharmacological mechanisms, the ROS scavenger N-acetylcysteine (NAC) (number S0077, Beyotime, China) and AMPK inhibitor Compound C (number P5499, Sigma-Aldrich, St. Louis, MO, USA) were used in the experiments. The IL-1*β* concentration in cell culture supernatants was measured by using the ELISA kit as noted above.

### 2.8. Cell Viability Assay, Oil Red O Staining, and Lipid Content Assay

After treatment, cell viability was determined by using the Cell Counting Kit-8 (number CK04, Dojindo Laboratories, Kumamoto, Japan) according to the manufacturer's instructions. Oil Red O staining and lipid content were measured as previously described [34].

### 2.9. Oxidative Stress Measures

Intracellular ROS and mitochondrial ROS levels were detected by using DCFH-DA (number S0033, Beyotime, China) and MitoSOX (number M36008, Invitrogen, Carlsbad, CA) according to the manufacturer's instructions. Fluorescence was measured using a Tecan Infinite 200 PRO microplate reader (Tecan Group Ltd., Mannedorf, Switzerland).

### 2.10. RNA Interference

For RNA interference experiments, hepatocytes were transfected with 20 nM scramble or AMPK short interfering RNA (siRNA) for AMPK*α*1/2 using the HiPerFect transfection reagent (number 301705, Qiagen, Valencia, CA, USA), according to the manufacturer's instructions. siRNAs were purchased from RiboBio (Guangzhou, China) as we described previously [26].

### 2.11. Immunoblot Analysis

Protein samples were extracted from liver tissues and cultured cells as previously reported [28]. Equal amounts of protein (30–40 *μ*g) were separated by 8%–15% SDS-PAGE and then transferred onto PVDF membranes. For immunoblot analysis of IL-1*β* levels in the cell culture medium, supernatants were collected for protein extraction as previously reported [35]. The following primary antibodies were used: anti-IL-1*β* (number 12242), anti-AMPK (number 2532), anti-phospho-AMPK Thr^172^ (number 2535), anti-phospho-GSK3*β* Ser^9^ (number 5558), anti-acetyl coenzyme A carboxylase (ACC, number 3676), anti-ACC Ser^79^ (number 3661) (Cell Signaling Technology, Beverly, MA, USA), anti-phospho-Akt Ser^473^ (number 2118-1) (Epitomics, Burlingame, CA, USA), anti-caspase-1 (number 22915-1-AP), anti-NLRP3 (number 19771-1-AP), anti-GSK3*β* (number 22104-1-AP) (Proteintech Group, Chicago, IL, USA), anti-TXNIP (number sc-67134) (Santa Cruz Biotechnology, Santa Cruz, CA, USA), and anti-Akt (number A0001)(ABclonal Biotech Co. Ltd., Cambridge, MA, USA). Goat anti-rabbit (number A21020) and goat anti-mouse (number A21010) secondary antibodies were purchased from Abbkine (Redlands, CA, USA).

### 2.12. Statistical Analysis

All data are expressed as the means ± s.e.m. form at least three independent experiments. SPSS 13.0 was used for statistical analysis. Unpaired Student's *t*-test was performed for analyzing individual group statistical comparison. Multiple group comparisons were evaluated by one-way ANOVA with post hoc testing. Values with *p* < 0.05 were considered as statistically significant.

## 3. Results

### 3.1. Salidroside Improves Body Weight and Glucose Control and Increases Insulin Sensitivity in HFD Mice

**Figure 1:**
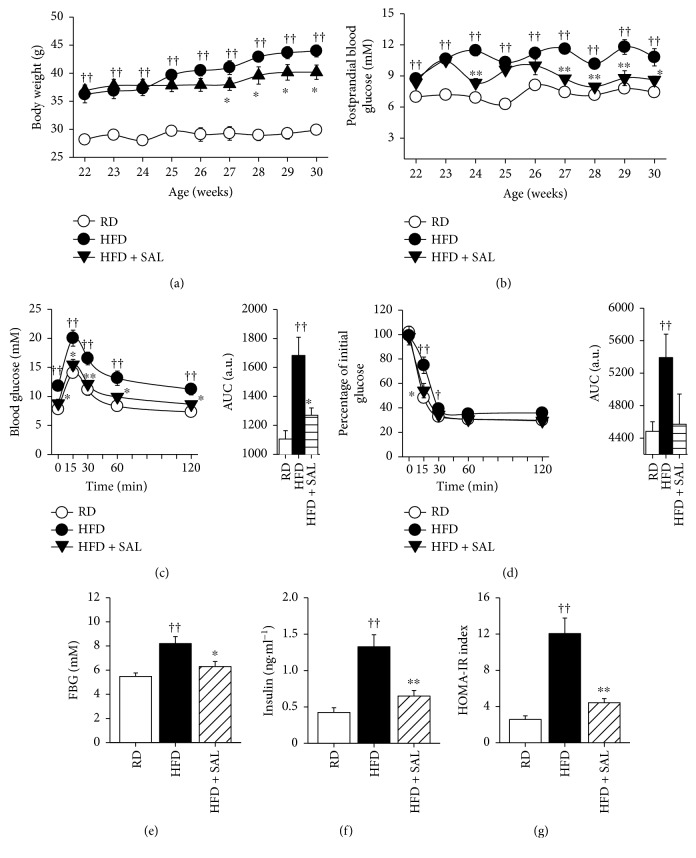
Effects of salidroside (SAL) on body weight, postprandial blood glucose, and insulin sensitivity in high-fat diet (HFD) mice. Vehicle or SAL (100 mg·kg^−1^·d^−1^) was administered orally to regular diet (RD) or HFD mice for 8 weeks, and body weight (a), postprandial blood glucose (b), intraperitoneal glucose tolerance test (IPGTT) (c), intraperitoneal insulin tolerance test (IPITT) (d), fasting blood glucose (FBG) (e), fasting serum insulin (f), and the homeostasis model assessment-insulin resistance (HOMA-IR) index (g) were detected. AUC: area under curve. ^†^ *P* < 0.05, ^††^ *P* < 0.01 versus RD mice treated with vehicle; ^∗^ *P* < 0.05, ^∗∗^ *P* < 0.01 versus HFD mice treated with vehicle. Values are means ± s.e.m. (*n* = 7).

In the current study, mice were fed either a RD or a HFD. Compared to RD mice, the body weight and blood glucose levels were increased in HFD mice. Moreover, HFD mice also exhibited impaired glucose tolerance and insulin sensitivity (Supplementary Figure 1), which are relevant features of NAFLD [36]. However, as shown in Figures 1(a) and 1(b), upon the treatment with 100 mg·kg^−1^·d^−1^ salidroside for 8 weeks, both of increased body weight and blood glucose levels were reduced in HFD mice. In addition, salidroside treatment also improved glucose tolerance and insulin sensitivity (Figures 1(c)–1(g)). Notably, we observed the similar results in salidroside-treated *db/db* mice in our previous study [25]. These results suggest that salidroside protects mice against HFD-induced obesity and insulin resistance.

### 3.2. Salidroside Improves Lipid Profiles and Alleviates the Hepatic Steatosis in HFD Mice

**Figure 2:**
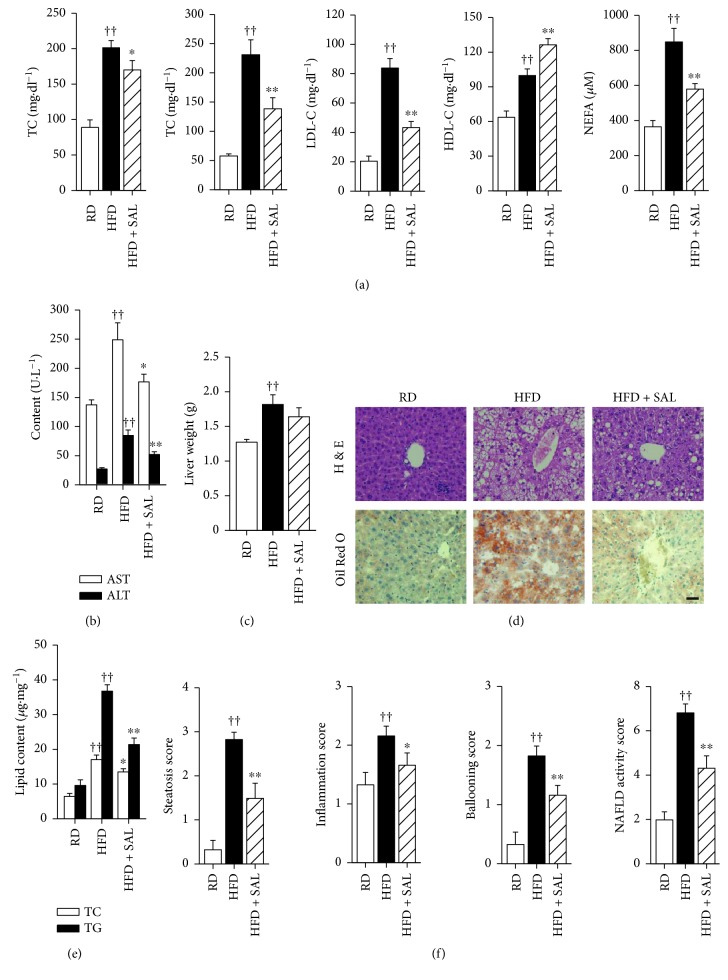
Salidroside (SAL) improves serum lipid profiles and alleviates hepatic steatosis in HFD mice. After treatment with SAL (100 mg·kg^−1^·d^−1^) for 8 weeks, serum samples and liver tissues were obtained from RD and HFD mice. The levels of serum TC, TG, HDL-C, LDL-C, and NEFA (a) and the levels of AST and ALT (b) were measured, and the liver weight (c) and hepatic lipid content (e) were detected. Liver sections were prepared, hematoxylin-eosin (H & E) staining and Oil Red O staining (d) were carried out, and steatosis, inflammation, ballooning, and NAFLD activity (f) were scored. Scale bar = 200 *μ*m. ^††^ *P* < 0.01 versus RD mice treated with vehicle; ^∗^ *P* < 0.05, ^∗∗^ *P* < 0.01 versus HFD mice treated with vehicle. Values are means ± s.e.m. ((a)–(c) and (f): *n* = 7; (d) and (e): *n* = 6).

The serum lipid profile of HFD mice is reported in Figure 2(a). It has been demonstrated that salidroside administration decreased the levels of TC, TG, LDL-C, and NEFA and increased the level of HDL-C. Similarly, biochemical analyses revealed that an increase in ALT and AST levels in HFD mice was reduced by salidroside treatment (Figure 2(b)). Here, salidroside treatment showed no influence on liver weight in HFD mice compared to vehicle control (Figure 2(c)). However, a significant lipid accumulation could be observed in the liver tissue of HFD mice, which was diminished by salidroside treatment (Figures 2(d) and 2(e)). Moreover, the histopathological analysis revealed that the score on steatosis, inflammation, ballooning, and NAFLD activity was higher in HFD mice, and salidroside administration tends to counteract this effect, as shown in Figure 2(f). Taken together, these results indicate that salidroside effectively corrects the HFD-induced dyslipidemia and hepatic steatosis in mice.

### 3.3. Salidroside Alleviates Oxidative Stress and Inflammatory Reaction in HFD Mice

**Figure 3:**
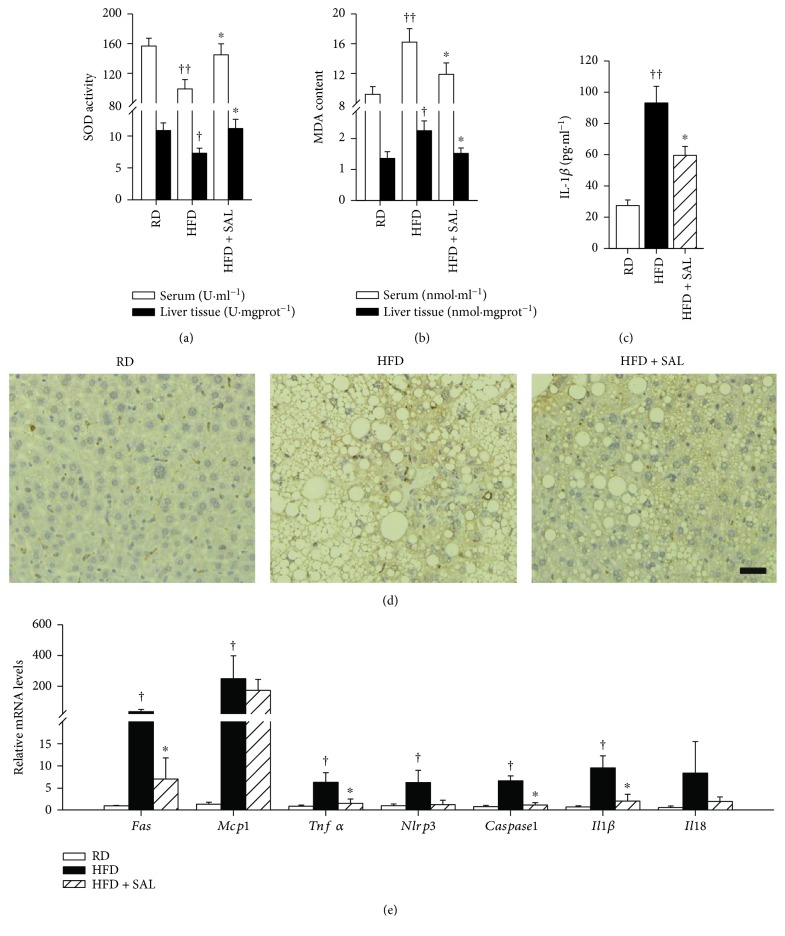
Salidroside (SAL) alleviates oxidative stress and inflammatory reaction in HFD mice. After treatment with SAL (100 mg·kg^−1^·d^−1^) for 8 weeks, serum and liver tissues were obtained from RD and HFD mice. The SOD activity (a), MDA content (b) in serum/liver tissues, and IL-1*β* concentration (c) in serum were measured. Liver sections were prepared for CD68 staining (d). Total RNA was extracted from liver tissue, and the gene expression levels of *Fas*, *Mcp1*, *Tnfα*, *Nlrp3*, *caspase1*, *Il1β*, and *Il18* were analyzed (e). Scale bar = 200 *μ*m. ^†^ *P* < 0.05, ^††^ *P* < 0.01 versus RD mice treated with vehicle; ^∗^ *P* < 0.05 versus HFD mice treated with vehicle. Values are means ± s.e.m. ((a)–(c): *n* = 7; (d) and (e): *n* = 4).

**Figure 4:**
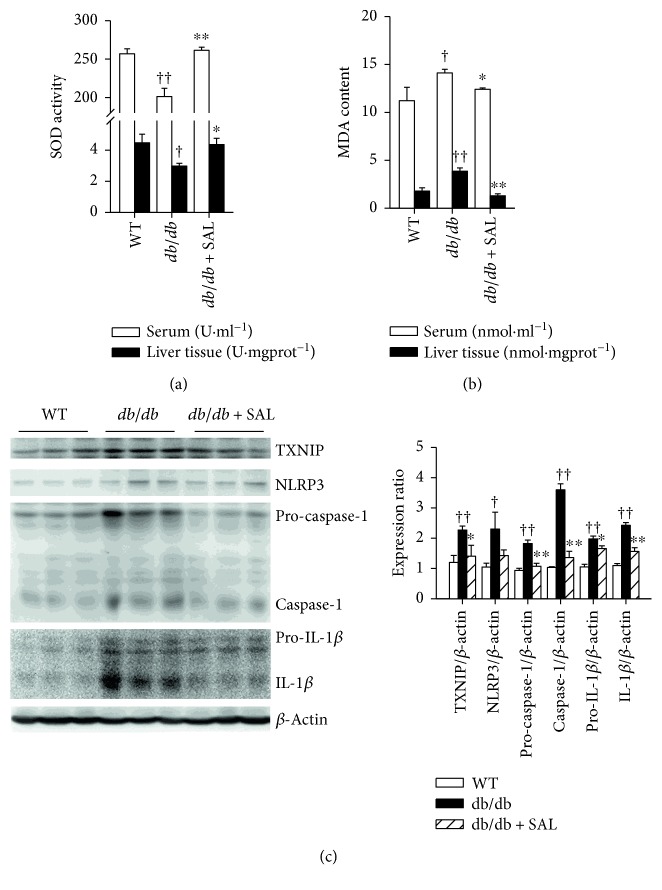
Salidroside (SAL) alleviates oxidative stress and suppresses NLRP3 inflammasome activation in *db/db* mice. After treatment with SAL (100 mg·kg^−1^·d^−1^) for 8 weeks, serum and liver tissues were obtained from wild-type (WT) and *db/db* mice. The SOD activity (a) and MDA content (b) in serum and liver tissues were measured. The expressions levels of TXNIP, NLRP3, caspase-1, and IL-1*β* in the liver tissues were detected by immunoblot (c). ^†^ *P* < 0.05, ^††^ *P* < 0.01 versus WT treated with vehicle; ^∗^ *P* < 0.05, ^∗∗^ *P* < 0.01 versus *db/db* mice treated with vehicle. Values are means ± s.e.m. ((a) and (b): *n* = 6; (c): *n* = 3).

The levels of nonenzymatic ROS scavengers SOD and oxidative stress marker MDA were evaluated *in vivo* with the aim to investigate the effect of salidroside on oxidative stress. As shown in Figures 3(a) and 3(b), HFD feeding caused a decrease in SOD activity and increase in MDA levels in serum and liver tissues, and both effects were alleviated by salidroside treatment. Consistently, similar effects of salidroside were also observed in *db/db* mice (Figures 4(a) and 4(b)). Interestingly, the results of biochemical analysis demonstrated that the concentration of IL-1*β* was significantly increased in HFD mice, but reduced upon salidroside treatment (Figure 3(c)). In addition, the results of CD68 staining indicated that more macrophages were scattered throughout liver tissues in HFD mice, and this result was improved by salidroside (Figure 3(d)). Furthermore, qRT-PCR revealed that salidroside treatment suppressed gene expressions of *Fas*, *Tnfα*, *Nlrp3*, *caspase1*, and *Il1β* in the liver, which were highly expressed in HFD mice compared to their RD counterparts (Figure 3e). *Fas* gene involves lipogenesis, and its increased expression may accelerate the ectopic deposition of TG [37]. Elevated expression of *Tnfα*, *Nlrp3*, *caspase1*, and *Il1β* revealed that proinflammatory factor production and NLRP3 inflammasome activation may be affected by salidroside treatment. Our results therefore demonstrate that salidroside exerts intervening effects on oxidative stress and NLRP3 inflammasome activation in NLFLD.

### 3.4. Salidroside Increases Phosphorylation of AMPK and ACC and Suppresses Activation of NLRP3 Inflammasome in the Liver

**Figure 5:**
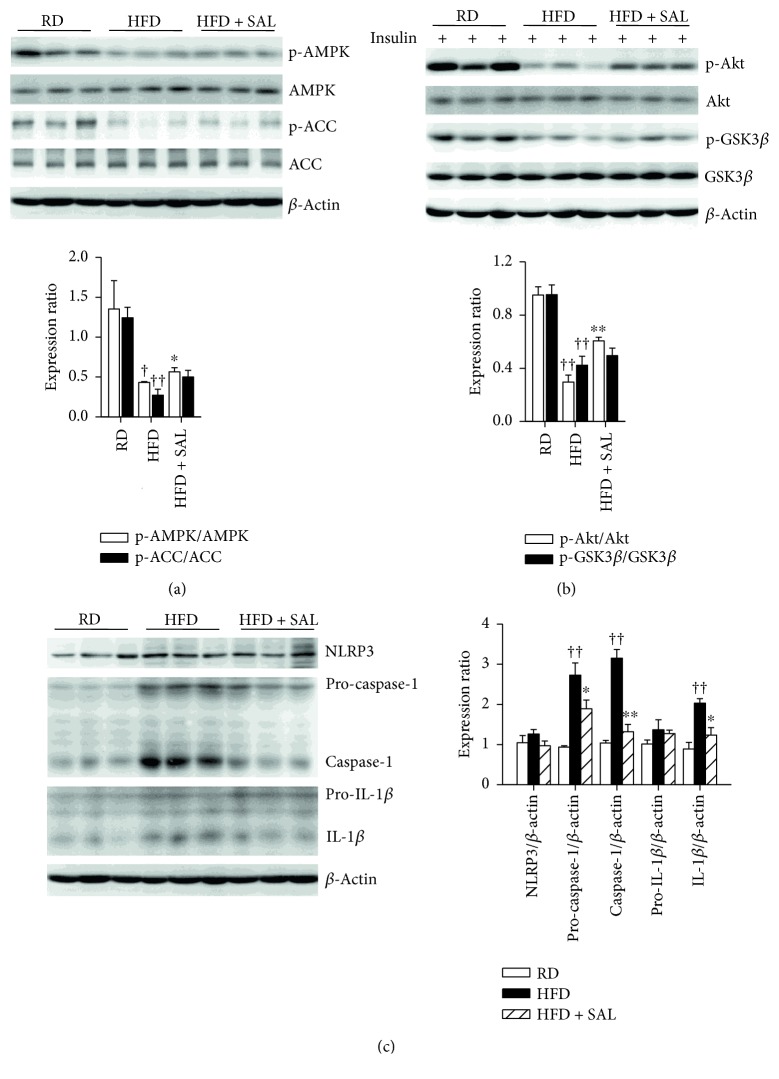
Salidroside (SAL) increases phosphorylation of AMPK, ACC, Akt, and GSK3*β* and suppresses activation of NLRP3 inflammasome in HFD mice. After treatment with SAL (100 mg·kg^−1^·d^−1^) for 8 weeks, liver tissues were obtained from RD and HFD mice. For the assessment of insulin sensitivity *in vivo*, mice were stimulated with 0.75 mU·g^−1^ insulin for 10 min after a 12 h fast before sacrificed. The phosphorylation of AMPK, ACC (a), Akt, and GSK3*β* (b) and the activation of NLRP3 inflammasome (c) were analyzed by immunoblot. ^†^ *P* < 0.05, ^††^ *P* < 0.01 versus RD mice treated with vehicle; ^∗^ *P* < 0.05, ^∗∗^ *P* < 0.01 versus HFD mice treated with vehicle. Values are means ± s.e.m. (*n* = 3).

As shown in Figures 5(a) and 5(b), the oral administration of salidroside for 8 weeks was capable of reversing the decreased phosphorylation of AMPK and ACC, as well as the reduced phosphorylation of Akt and GSK3*β* in the liver. Moreover, our results also revealed that the levels of activated form of caspase-1 and IL-1*β* were both increased in HFD mice (Figure 5(c)) and *db/db* mice (Figure 4(c)), which suggested that NLRP3 inflammasome activation was increased. However, after treatment with salidroside, the cleaved caspase-1 and IL-1*β* were both decreased in HFD and *db/db* mice, and the expression level of pro-IL-1*β* was reduced in *db/db* mice. Thus, these results provide first evidences that salidroside can inhibit NLRP3 inflammasome activation in NAFLD and type 2 diabetes.

### 3.5. Salidroside Improves Abnormal Lipid Accumulation and AMPK/ACC Phosphorylation in a Dose-Dependent Manner In Vitro

**Figure 6:**
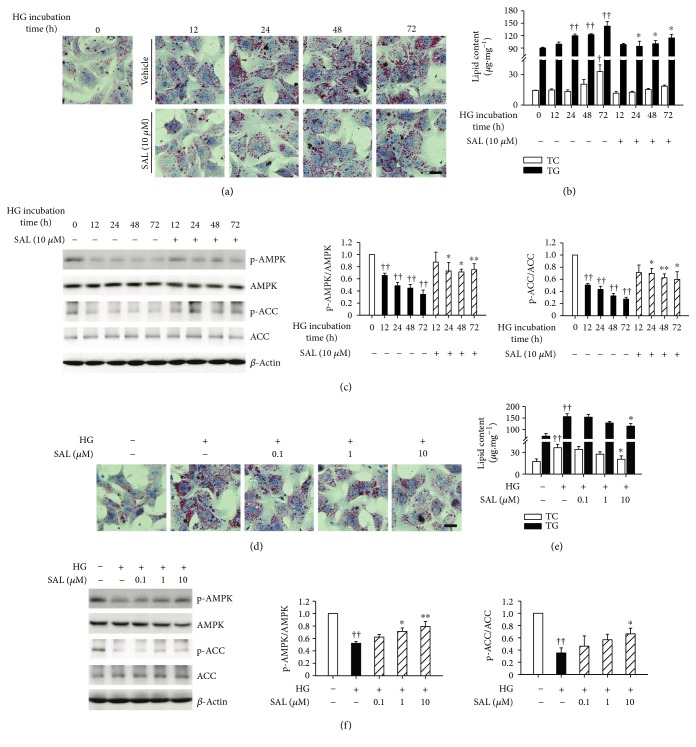
Salidroside (SAL) improves abnormal lipid accumulation and AMPK activity in a dose-dependent manner in hepatocytes. After cultured overnight in the serum-free medium which contains normal glucose (NG, 5.5 mM), primary mouse hepatocytes were incubated in the serum-free medium which contains 30 mM glucose and 100 nM insulin (HG) and treated with 10 *μ*M SAL for the indicated periods of time (0–72 h), or treated with SAL (0.1, 1, 10 *μ*M) for 72 h. The Oil Red O staining (a, d) and lipid content detection (b, e) were carried out. Protein sample was extracted from hepatocytes, and the phosphorylation of AMPK and ACC (c, f) was analyzed by immunoblot. Scale bar = 200 *μ*m. ^†^ *P* < 0.05, ^††^ *P* < 0.01 versus NG; ^∗^ *P* < 0.05, ^∗∗^ *P* < 0.01 versus HG. Values are means ± s.e.m. ((a) and (d): *n* = 3; (b), (c), (e), and (f): *n* = 4).

Oil Red O staining and lipid content analysis demonstrated that lipid accumulation was increased in hepatocytes cultured under high glucose plus insulin condition from 24 to 72 h (Figures 6(a) and 6(b)). Notably, such culture conditions could mimic hyperglycemia and insulin resistance and cause superfluous lipid deposition *in vivo*, according to previous reports [25, 33]. However, salidroside treatment inhibited lipid deposition and increased the phosphorylation of AMPK and ACC in hepatocytes in a dose-dependent manner (Figures 6(a)–6(f)).

### 3.6. Salidroside Improves Insulin Sensitivity and Suppresses NLRP3 Inflammasome Activation In Vitro

**Figure 7:**
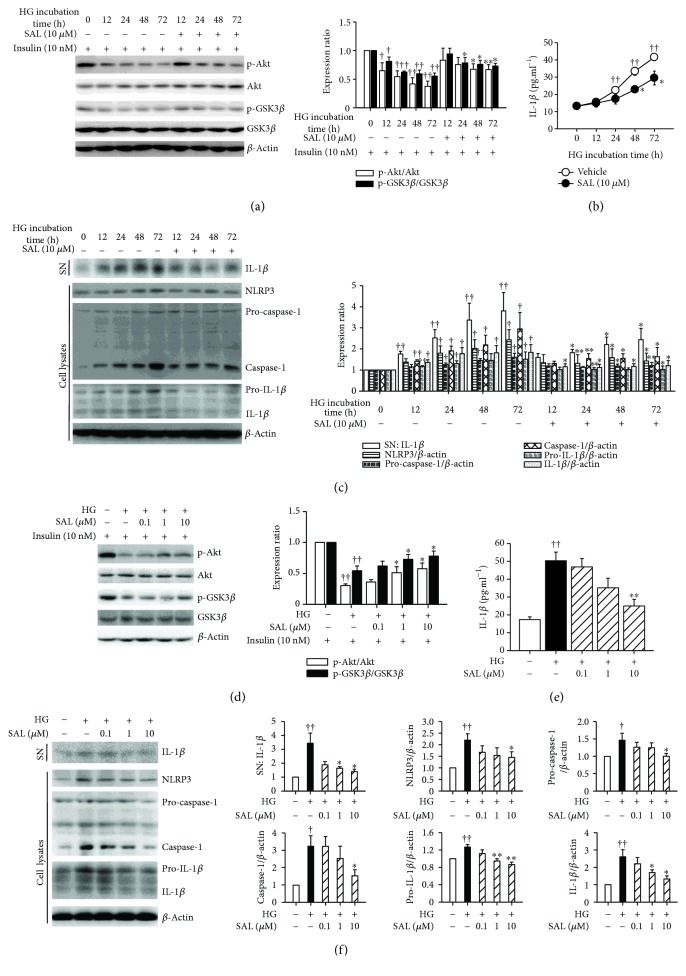
Salidroside (SAL) improves insulin sensitivity and suppresses NLRP3 inflammasome activation in hepatocytes. After cultured overnight in the serum-free medium which contains normal glucose (NG, 5.5 mM), primary mouse hepatocytes were incubated in the serum-free medium which contains 30 mM glucose and 100 nM insulin (HG) and treated with 10 *μ*M SAL for the indicated periods of time (0–72 h), or treated with SAL (0.1, 1, and 10 *μ*M) for 72 h. For assessment of insulin sensitivity *in vitro*, hepatocytes were treated as indicated, the medium was removed, and cells were incubated in fresh serum-free DMEM containing insulin (10 nM) for 20 min before protein samples were extracted. Protein sample were extracted from hepatocytes or supernatant (SN). The phosphorylation of Akt and GSK3*β* (a, d) and the activation of NLRP3 inflammasome (c, f) were analyzed by immunoblot. The supernatant IL-1*β* concentration (b, e) was measured by ELISA method. ^†^ *P* < 0.05, ^††^ *P* < 0.01 versus NG; ^∗^ *P* < 0.05, ^∗∗^ *P* < 0.01 versus HG. Values are means ± s.e.m. ((a), (c), (d), and (f): *n* = 4; (b) and (e): *n* = 3).

We further investigated the effects of salidroside on insulin sensitivity and NLRP3 inflammasome activation *in vitro*. As shown in Figures 7(a)–7(f), in high glucose plus insulin condition, the levels of insulin-stimulated phosphorylation of Akt and GSK3*β* were blunted and the active form of caspase-1 and IL-1*β* was increased, respectively, indicating that the insulin sensitivity was impaired and NLRP3 inflammasome activation was enhanced. Conversely, our results revealed that the treatment with salidroside restored the insulin-stimulated phosphorylation of Akt and GSK3*β* and reduced the levels of caspase-1 and IL-1*β*. Our results revealed that neither high glucose plus insulin condition nor salidroside incubation had cytotoxic effects on hepatocytes (Supplementary Figure 2(a)). In addition, compared to glucose, the same concentration of mannitol (30 mM) exhibited no effects on lipid accumulation and AMPK and ACC phosphorylation in hepatocytes, thus excluding the potential influence of osmotic pressure (Supplementary Figures 2(b) and 2(c)). Even in this case, the potential influence of osmotic pressure on insulin sensitivity or NLRP3 inflammasome activation in hepatocytes was excluded by using mannitol (Supplementary Figures 2(d) and 2(e)). Overall, these results demonstrated that salidroside can protect hepatocytes against metabolic stress related to anomalies in insulin signaling transduction and NLRP3 inflammasome activation.

### 3.7. Salidroside Protects Hepatocytes against ROS Overproduction under High Glucose Plus Insulin Condition

**Figure 8:**
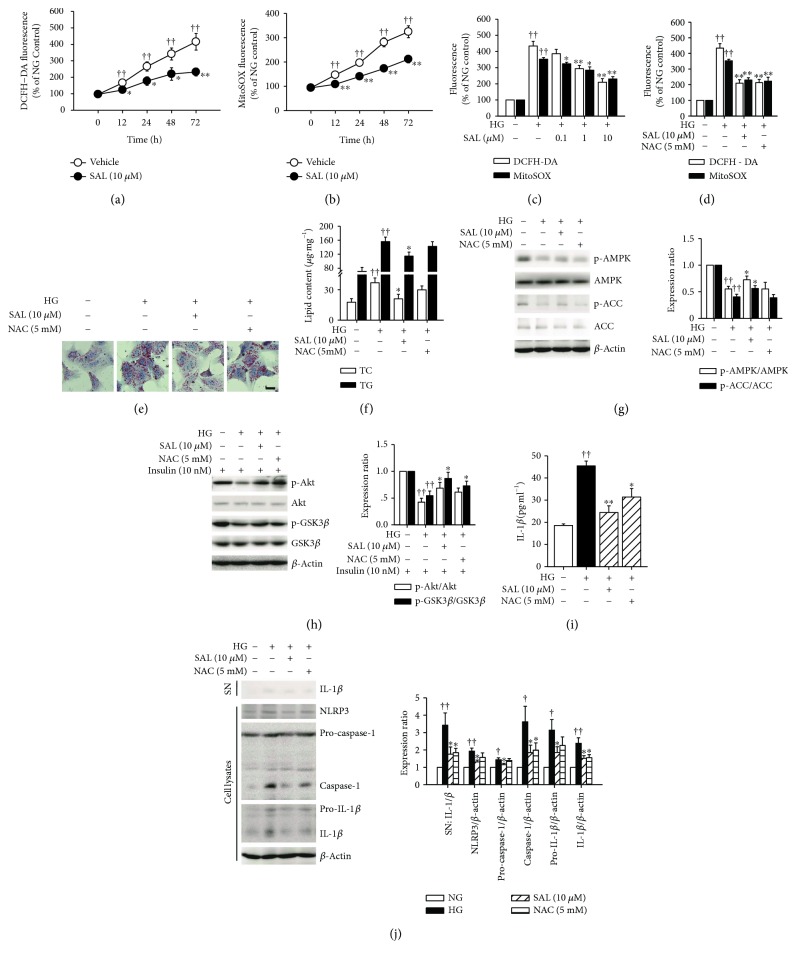
Salidroside (SAL) protects hepatocytes against ROS overproduction. After cultured overnight in the serum-free medium which contains normal glucose (NG, 5.5 mM), hepatocytes were incubated in the serum-free medium which contains 30 mM glucose and 100 nM insulin (HG) and treated with 10 *μ*M SAL for the indicated periods of time (0–72 h), or treated with SAL (0.1, 1, 10 *μ*M) and N-acetylcysteine (NAC, 5 mM) for 72 h. The ROS levels were detected using DCF-DA and MitoSOX as indicated (a–d). The Oil Red O staining (e) and lipid content analysis (f) were performed. Scale bar = 200 *μ*m. Protein sample was extracted from hepatocytes or supernatant (SN). For assessment of insulin sensitivity *in vitro*, hepatocytes were treated as indicated, the medium was removed, and cells were incubated in fresh serum-free DMEM containing insulin (10 nM) for 20 min before protein samples were extracted. The phosphorylation of AMPK, ACC (g), Akt, and GSK3*β* (h) and the activation of NLRP3 inflammasome (j) were analyzed by immunoblot. The supernatant IL-1*β* concentration was measured by ELISA method (i). ^†^ *P* < 0.05, ^††^ *P* < 0.01 versus NG; ^∗^ *P* < 0.05, ^∗∗^ *P* < 0.01 versus HG. Values are means ± s.e.m. ((a)–(d), (f)–(h), and (j): *n* = 4; (e) and (i): *n* = 3).

Since ROS are essential for NLRP3 inflammasome activation [38, 39], we measured the levels of cellular ROS and mitochondrial ROS by using fluorescent probes DCFH-DA and MitoSOX. As shown in Figures 8(a)–8(c), salidroside suppressed the ROS overproduction in hepatocytes exposed to high glucose plus insulin. To verify whether salidroside-induced ROS decreasing was necessary for its therapeutic effect on NAFLD, hepatocytes were incubated with the ROS scavenger NAC. Compared to salidroside, NAC exerted similar effects on ROS production, insulin sensitivity, and NLRP3 inflammasome activation in hepatocytes (Figures 8(d), 8(h), 8(i), and 8(j)). However, NAC incubation had no influences on either lipid deposition or AMPK and ACC phosphorylation in hepatocytes (Figures 8(e)–8(g)).

### 3.8. AMPK Activation May Account for the Inhibitory Effects of Salidroside on TXNIP/NLRP3 Signaling Activation

**Figure 9:**
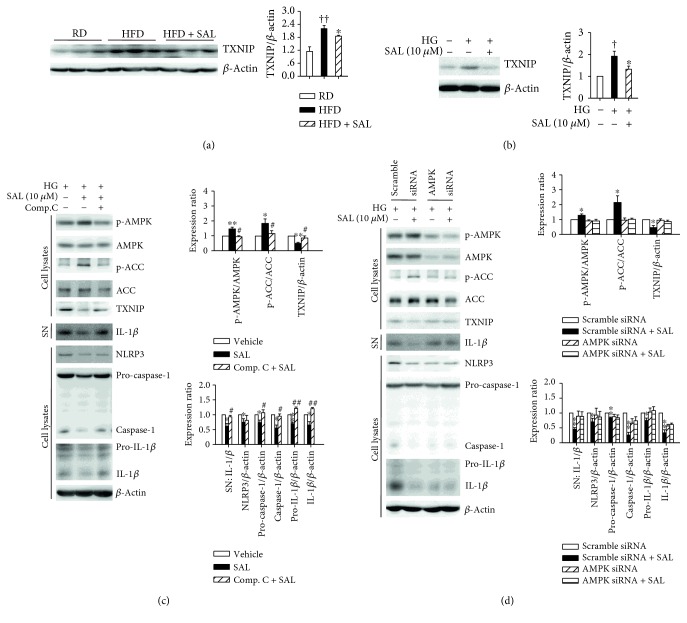
AMPK activation is necessary for salidroside- (SAL-) mediated regulation on TXNIP/NLRP3 pathway. After treatment with SAL (100 mg·kg^−1^·d^−1^) for 8 weeks, the liver tissues were obtained from RD and HFD mice. The expression levels of TXNIP (a) were analyzed by immunoblot. ^††^ *P* < 0.01 versus RD mice treated with vehicle; ^∗^ *P* < 0.05 versus HFD mice treated with vehicle. Values are means ± s.e.m. (*n* = 3). After cultured overnight in the serum-free medium which contains normal glucose (NG, 5.5 mM), hepatocytes were incubated in the serum-free medium which contains 30 mM glucose and 100 nM insulin (HG) and treated with 10 *μ*M SAL for 72 h. The expression levels of TXNIP (b) were detected by immunoblot. ^†^ *P* < 0.05 versus NG; ^∗^ *P* < 0.05 versus HG. Values are means ± s.e.m. (*n* = 4). After cultured overnight in the serum-free medium which contains NG, hepatocytes were incubated in the HG and treated with 10 *μ*M Compound C (Comp. C) for 30 min before coincubated with SAL (10 *μ*M) for 72 h. Protein sample was extracted from hepatocytes or supernatant (SN). The levels of p-AMPK, p-ACC, and TXNIP and the activation of NLRP3 inflammasome were analyzed by immunoblot (c). ^∗^ *P* < 0.05, ^∗∗^ *P* < 0.01 versus HG plus vehicle; ^#^ *P* < 0.05, ^##^ *P* < 0.01 versus HG plus SAL. Values are means ± s.e.m. (*n* = 4). Hepatocytes were transfected with 20 nM AMPK*α*1/*α*2 or scrambled siRNA for 24 h, then incubated in HG and treated with 10 *μ*M SAL for 48 h as indicated. The phosphorylation of AMPK and ACC and the activation of NLRP3 inflammasome were analyzed by immunoblot (d). ^∗^ *P* < 0.05, ^∗∗^ *P* < 0.01 versus scramble siRNA without SAL. Values are means ± s.e.m. (*n* = 4).

TXNIP has been suggested as mediator of high glucose-induced NLRP3 inflammasome activation [17, 40]. We detected TXNIP expression levels both *in vivo* and *in vitro*. Interestingly, compared to controls, TXNIP expression levels were significantly increased in liver tissues of HFD mice and *db/db* mice. Salidroside administration markedly suppressed the overexpression of TXNIP (Figures 4(c) and 9(a)). Moreover, the *in vitro* assay revealed that the TXNIP level was increased in hepatocytes exposed to high glucose plus insulin and reversed by salidroside (Figure 9(b)). According to a previous study [41], we speculated that salidroside-induced AMPK activation might regulate TXNIP expression. Thus, we explored the role of AMPK activation in salidroside-induced effects on TXNIP expression by using AMPK inhibitor Compound C and AMPK siRNA. As shown in Figure 9(c), the effects of salidroside on AMPK and ACC phosphorylation, TXNIP expression, and NLRP3 inflammasome activation disappeared after coincubation with Compound C. Furthermore, AMPK siRNA transfection also extinguished the effects of salidroside in hepatocytes (Figure 9(d)). Thus, AMPK activation is necessary for regulating the effects of salidroside on TXNIP/NLRP3 signaling.

## 4. Discussion

NAFLD is a widely recognized pathology and recently became the most common cause of chronic liver diseases, a spectrum of liver pathologies encompassed by the initial early stage of steatosis to steatohepatitis and fibrosis [42]. Factors and stressors linked to NAFLD include obesity, hyperlipidemia, insulin resistance, and type 2 diabetes [43].

Recently, we reported both hypolipidemic and hypoglycemic effects of salidroside on type 2 diabetes [25]. We found that salidroside-stimulated AMPK activation could reduce lipid accumulation in muscle and liver tissues of *db/db* mice, suggesting that salidroside may be used for the treatment of NAFLD [25]. Yang et al. [36] and Li et al. [44] reported that salidroside could prevent liver injury in the rat or mice NASH model induced by HFD feeding, and these findings raised that the antioxidant and hepatic insulin signaling regulation are involved in the action of salidroside, respectively. However, whether or not salidroside is suitable for NAFLD, treatment and mechanisms underlying its effects have not been fully elucidated yet. In the present study, we investigated the pharmacological effects of salidroside both *in vivo* and *in vitro*, showing that salidroside has potential for NAFLD treatment, and its action is associated with the regulation of oxidative stress and AMPK-dependent TXNIP/NLRP3 pathway.

Compared with control RD mice, HFD mice developed a stably higher body weight, an abnormal blood glucose fluctuation, and an impaired glucose tolerance. Salidroside administration effectively decreased body weight and blood glucose levels and improved insulin sensitivity in HFD mice. Furthermore, salidroside treatment also ameliorated dyslipidemia and suppressed lipid deposition in the liver. Biochemical analyses revealed that salidroside treatment corrected deficiencies in HFD mice, such as an increase in levels of AST, ALT, MDA, and IL-1*β* and a decrease in SOD and AMPK activity. Moreover, salidroside caused a dose-dependent decrease in lipid accumulation and phosphorylation of AMPK and ACC and also improved insulin sensitivity in cultured hepatocytes. Thus, these observations indicate that salidroside treatment can ameliorate NAFLD.

A previous study reported that compared with controls, the activation of NLRP3 inflammasome in liver tissues in NASH was enhanced [45], and NLRP3 inflammasome activation promoted the development of NASH toward to fibrosis [46]. Generally, once NLRP3 inflammasome is activated, the pro-caspase-1 is subject to auto-cleavage with the formation of the active caspase-1, which is able to process cytokine proforms such as pro-IL-1*β* and pro-IL-18 to generate the active IL-1*β* and IL-18, respectively [10]. Accumulated evidences suggested that IL-1*β* was deeply involved in the pathogenesis of NAFLD and NASH [6, 47, 48]. For example, compared to wild-type mice, *Nlrp3^−/−^* or *caspase1^−/−^* were protected from feeding-induced hepatomegaly, hepatic steatosis, inflammation, and liver fibrosis [8, 46]. Similarly, in *Tlr9^−/−^* mice and *Il1βr^−/−^* mice, the IL-1*β* treatment-induced lipid accumulation and resulting cell death were both alleviated compared to the wild type, respectively [48]. Notably, our study demonstrated that salidroside treatment dramatically suppressed the superfluous macrophage scatter and NLRP3 inflammasome activation in the liver in HFD mice. Interestingly, a similar effect of salidroside on NLRP3 inflammasome was also observed in the liver of *db/db* mice. Heretofore, our previous study revealed that *db/db* mice exhibited characteristics of hepatic lipidosis even under RD feeding [25]. This finding indicated that the inhibition of NLRP3 inflammasome activation may be involved in the beneficial action of salidroside in metabolic disorder, such as NAFLD and type 2 diabetes.

Increasing evidences suggested that TXNIP links oxidative stress to NLRP3 inflammasome activation [11, 17, 38, 40]. TXNIP is an NLRP3-binding protein, and the C-terminal domain of TXNIP and the NACHT domain of NLRP3 can interact, leading to the activation of NLRP3 inflammasome [17]. Under oxidative stress conditions, TXNIP is released from TRX after its oxidation by ROS. On the other hand, impaired glucose homeostasis not only triggers the elevation of ROS [49] but also enhances TXNIP expression via regulation of the transcription factor, carbohydrate response element-binding protein [50]. Thus, TXNIP represents a promising new therapeutic target for many pathologies related to metabolic diseases, including diabetic retinopathy [51] and retinal neurodegenerative diseases [15]. In this framework, we evaluated the effects of salidroside on TXNIP levels *in vivo* and *in vitro*. Our results revealed that salidroside decreased protein levels both in liver tissue and cultured hepatocytes. Moreover, upon salidroside treatment, the anomalous SOD activity and MDA levels in mice were restored. Salidroside also suppressed the increase in cellular ROS in hepatocytes exposed to high glucose plus insulin. Thus, salidroside decreased oxidative stress and TXNIP levels both *in vivo* and *in vitro*.

Many previous studies mentioned the antioxidative stress property of salidroside both *in vivo* and *in vitro* [36, 42, 52]. However, mechanisms underlying this property have not been fully understood. Based on our previous studies, we concluded that salidroside may target mitochondria to inhibit respiratory chain complex I [25] and to stimulate mitochondrial biogenesis [27], therefore decreasing the rate of superoxide production [26]. In this study, we used NAC, a ROS scavenger, and we found that ROS production and NLRP3 activation were both suppressed. However, NAC treatment exerted no effect either on lipid deposition or on AMPK activity. Recently, a clinical trial demonstrated that single antioxidant therapy with NAC in NAFLD patients resulted in a decrease of serum ALT levels [53]. However, due to the lack of results on pathologic examination, there are still no sufficient data to support the use of antioxidant for treatment of NAFLD [54]. Our current findings disclose that the role of antioxidants is necessary for the action of salidroside, but insufficient for the treatment of NAFLD, and also that the regulation on AMPK activity and lipid metabolism in hepatocytes is crucial.

In our previous study, we clarified the pharmacological mechanism of salidroside on AMPK activation [25]. AMPK activation has been shown to decrease the protein level of TXNIP by regulating *Txnip* gene expression [55] or promoting TXNIP degradation [41]. These findings indicate that salidroside-induced AMPK activation may exert a key role in the regulation of TXNIP levels and NLRP3 inflammasome activation. To address this speculation, we blocked the AMPK activity by using both a specific inhibitor and siRNA. Interestingly, the inhibition of AMPK activity suppressed the action of salidroside on TXNIP expression and NLRP3 inflammasome activation. Taken together, these results demonstrate that AMPK activation may account for the inhibitory effects of salidroside on TXNIP/NLRP3 signaling activation. These findings suggest that salidroside may be suitable not only for NALFD but also for other inflammation and metabolic disorders.

Overall, salidroside exhibits therapeutic action on NAFLD. As shown in Supplementary Figure 3, we may speculate that mechanism underlying its effects involves antioxidative stress and regulation of AMPK-dependent TXNIP/NLRP3 signaling pathway, thus representing a potentially powerful target for NAFLD pharmacological purposes.

## Data Availability

The data used to support the findings of this study are available from the corresponding author upon request.

## Abbreviations

ACC: Acetyl coenzyme A carboxylase
ALT: Alanine transaminase
AMPK: AMP-activated protein kinase
AST: Aspartate transaminase
DAMPs: Damage-associated molecular patterns
FAS: Fatty acid synthase
HDL-C: HDL cholesterol
H & E: Hematoxylin-eosin
HFD: High-fat diet
HOMA-IR: Homeostasis model assessment-insulin resistance
IPGTT: I.P. glucose tolerance test
IPITT: I.P. insulin tolerance test
LDL-C: LDL cholesterol
MCP1: Monocyte chemoattractant protein 1
MDA: Malondialdehyde
NAC: N-Acetylcysteine
NAFLD: Nonalcoholic fatty liver disease
NASH: Nonalcoholic steatohepatitis
NEFA: Nonesterified fatty acid
NLRs: NOD-like receptors
PAMPs: Pathogen-associated molecular patterns
RD: Regular diet
ROS: Reactive oxygen species
siRNA: Short interfering RNA
TC: Total cholesterol
TG: Triglycerides
TNF-*α*: Tumor necrosis factor *α*
TRX: Thioredoxin
TXNIP: TRX-interacting protein.

## Supplementary Materials

Supplementary 1 Supplementary Figure 1: influences of HFD on body weight, postprandial blood glucose, and insulin sensitivity in mice. Mice were administered with RD or HFD for 14 weeks, and body weight (a), postprandial blood glucose (b), IPGTT (c), and IPITT (d) were determined. ^†^ *P* < 0.05, ^††^ *P* < 0.01 versus RD. Values are means ± s.e.m. (RD mice, *n* = 10; HFD mice, *n* = 20).

Supplementary 2 Supplementary Figure 2: salidroside (SAL) treatment had no influences on cell viability, as well as the effects of mannitol (MT) incubation on lipid accumulation, AMPK activity, insulin sensitivity, and NLRP3 inflammasome activation. After cultured overnight in the serum-free medium which contains normal glucose (NG, 5.5 mM), primary mouse hepatocytes were incubated in the serum-free medium which contains 30 mM glucose and 100 nM insulin (HG) and treated with vehicle or 10 *μ*M SAL for the indicated periods of time (0–72 h), and then cell viability (a) were assayed. After serum starvation overnight, primary mouse hepatocytes were incubated in the serum-free medium which contains HG or MT (30 mM) alone for 72 h, and then the Oil Red O staining (b) was carried out. Protein sample was extracted from hepatocytes or supernatant (SN). The phosphorylation of AMPK, ACC (c), Akt, and GSK3*β* (d) and the activation of NLRP3 inflammasome (e) were analyzed by immunoblot. For assessment of insulin sensitivity *in vitro*, hepatocytes were treated as indicated, the medium was removed, and cells were incubated in fresh serum-free DMEM containing insulin (10 nM) for 20 min before protein samples were extracted. Scale bar = 200 *μ*m. ^∗^ *P* < 0.05, ^∗∗^ *P* < 0.01 versus NG. Values are means ± s.e.m. ((a) and (b): *n* = 3; (c)–(e): *n* = 4).

Supplementary 3 Supplementary Figure 3: schematic diagram of SAL's action.
