# Supplementary figures and images for: Salidroside Attenuates High-Fat Diet-Induced Nonalcoholic Fatty Liver Disease via AMPK-Dependent TXNIP/NLRP3 Pathway
Source: Oxid Med Cell Longev. 2018 Jul 22;2018:8597897. doi: 10.1155/2018/8597897 (PMC6081551; doi:10.1155/2018/8597897)

Supplementary Figure 1


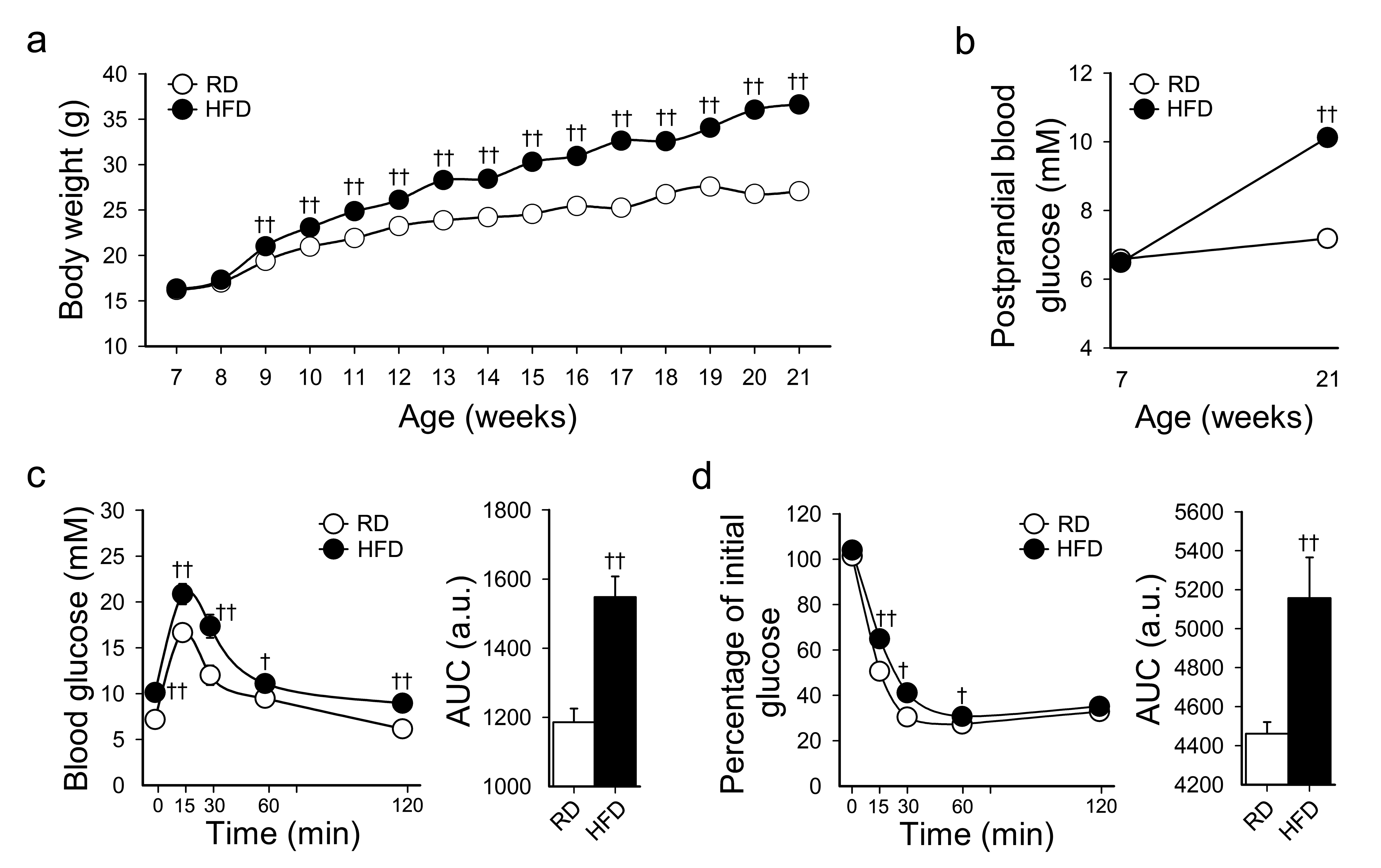

Supplement: Supplementary 1 — Supplementary Figure 1: influences of HFD on body weight, postprandial blood glucose, and insulin sensitivity in mice. Mice were administered with RD or HFD for 14 weeks, and body weight (a), postprandial blood glucose (b), IPGTT (c), and IPITT (d) were determined. † P < 0.05, †† P < 0.01 versus RD. Values are means ± s.e.m. (RD mice, n = 10; HFD mice, n = 20). [file 8597897.f1.docx]

Supplementary Figure 2


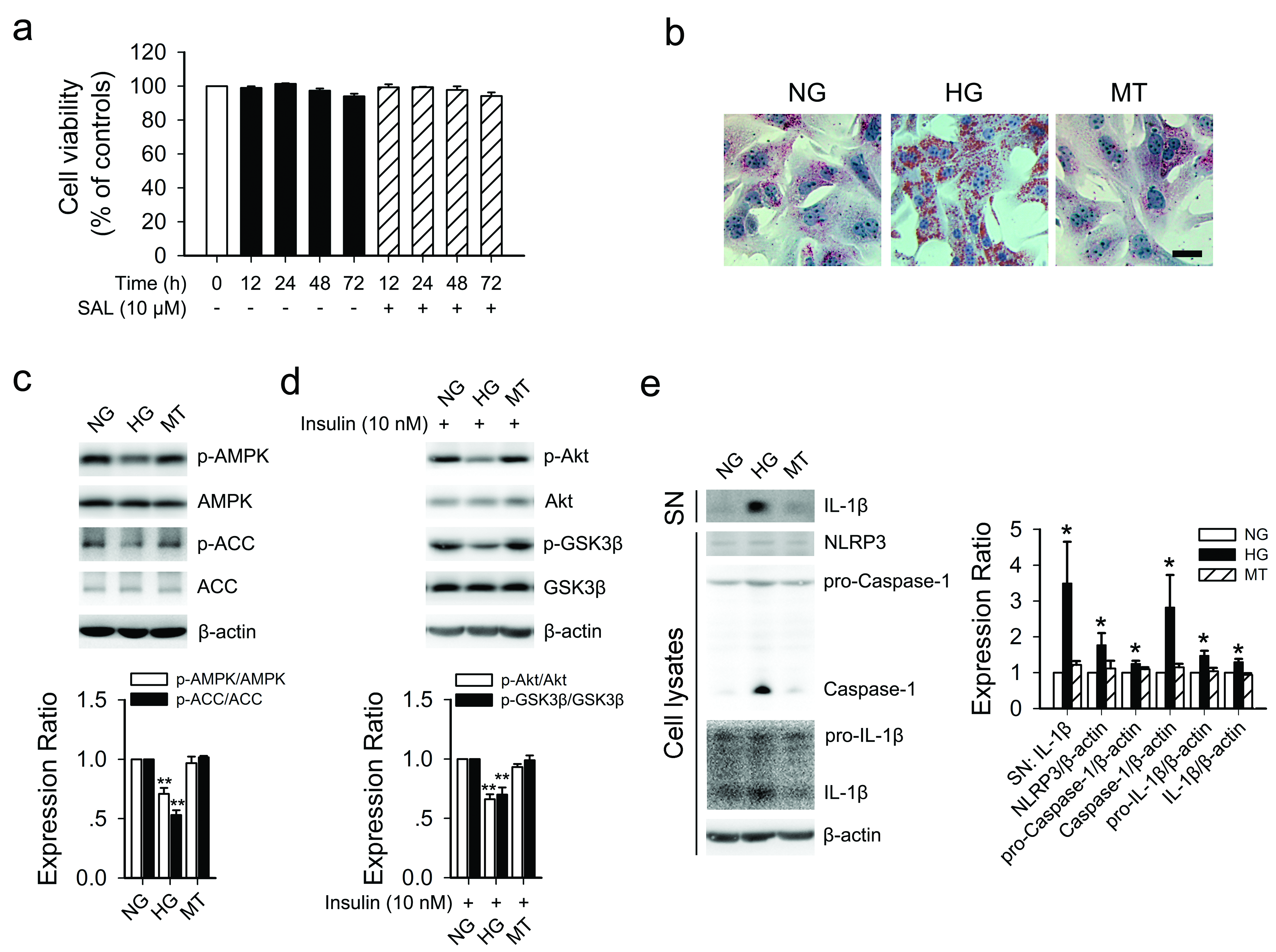

Supplement: Supplementary 2 — Supplementary Figure 2: salidroside (SAL) treatment had no influences on cell viability, as well as the effects of mannitol (MT) incubation on lipid accumulation, AMPK activity, insulin sensitivity, and NLRP3 inflammasome activation. After cultured overnight in the serum-free medium which contains normal glucose (NG, 5.5 mM), primary mouse hepatocytes were incubated in the serum-free medium which contains 30 mM glucose and 100 nM insulin (HG) and treated with vehicle or 10 μM SAL for the indicated periods of time (0–72 h), and then cell viability (a) were assayed. After serum starvation overnight, primary mouse hepatocytes were incubated in the serum-free medium which contains HG or MT (30 mM) alone for 72 h, and then the Oil Red O staining (b) was carried out. Protein sample was extracted from hepatocytes or supernatant (SN). The phosphorylation of AMPK, ACC (c), Akt, and GSK3β (d) and the activation of NLRP3 inflammasome (e) were analyzed by immunoblot. For assessment of insulin sensitivity in vitro, hepatocytes were treated as indicated, the medium was removed, and cells were incubated in fresh serum-free DMEM containing insulin (10 nM) for 20 min before protein samples were extracted. Scale bar = 200 μm. ∗ P < 0.05, ∗∗ P < 0.01 versus NG. Values are means ± s.e.m. ((a) and (b): n = 3; (c)–(e): n = 4). [file 8597897.f2.docx]

Supplementary Figure 3


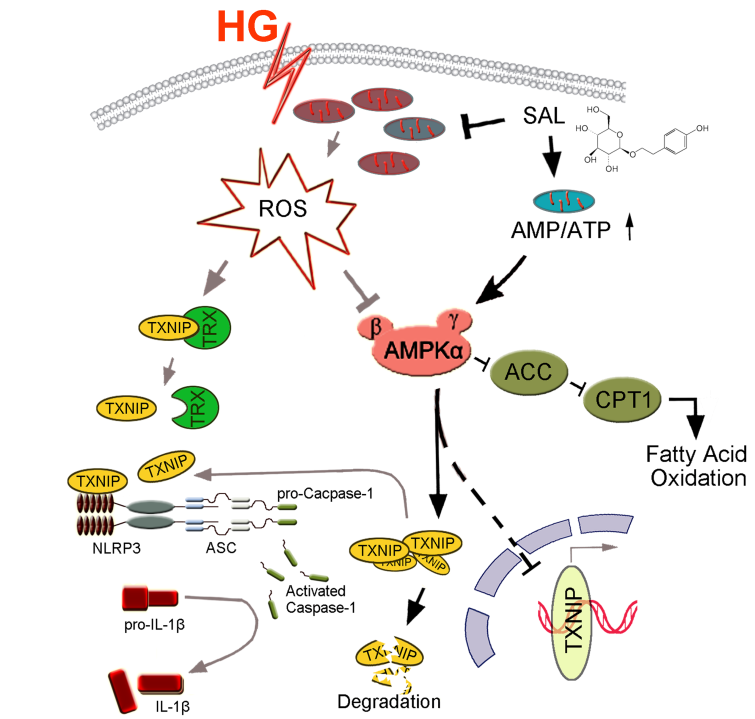

Supplement: Supplementary 3 — Supplementary Figure 3: schematic diagram of SAL's action. [file 8597897.f3.docx]
